# Aliphatic Polyurethane Elastomers Quaternized with Silane-Functionalized TiO_2_ Nanoparticles with UV-Shielding Features

**DOI:** 10.3390/polym13081318

**Published:** 2021-04-16

**Authors:** Lenuta Stroea, Andreea-Laura Chibac-Scutaru, Violeta Melinte

**Affiliations:** Polyaddition and Photochemistry Department, Petru Poni Institute of Macromolecular Chemistry, 41 A Grigore Ghica Voda Alley, 700487 Iasi, Romania; elenah@icmpp.ro (L.S.); andreea.chibac@icmpp.ro (A.-L.C.-S.)

**Keywords:** polyurethane, nanoparticle, nanocomposite, quaternization, mechanical properties, UV protective

## Abstract

The design of high-performance nanocomposites with improved mechanical, thermal or optical properties compared to starting polymers has generated special interest due to their use in a wide range of targeted applications. In the present work, polymer nanocomposites composed of polyurethane elastomers based on polycaprolactone or polycaprolactone/poly(ethylene glycol) soft segments and titanium dioxide (TiO_2_) nanoparticles as an inorganic filler were prepared and characterized. Initially, the surface of TiO_2_ nanoparticles was modified with (3-iodopropyl) trimethoxysilane as a coupling agent, and thereafter, the tertiary amine groups from polyurethane hard segments were quaternized with the silane-modified TiO_2_ nanoparticles in order to ensure covalent binding of the nanoparticles on the polymeric chains. In the preparation of polymer nanocomposites, two quaternization degrees were taken into account (1/1 and 1/0.5 molar ratios), and the resulting nanocomposite coatings were characterized by various methods (Fourier transform infrared spectroscopy, X-ray diffraction, scanning electron microscopy, contact angle, thermogravimetric analysis, dynamic mechanical thermal analysis). The mechanical parameters of the samples evaluated by tensile testing confirm the elastomeric character of the polyurethanes and of the corresponding composites, indicating the obtaining of highly flexible materials. The absorbance/transmittance measurements of PU/TiO_2_ thin films in the wavelength range of 200–700 nm show that these partially block UV-A radiation and all UV-B radiation from sunlight and could possibly be used as UV-protective elastomeric coatings.

## 1. Introduction

Thermoplastic polyurethanes (TPUs) are an important category of polymeric materials synthesized by step-growth polymerization of polyols as soft segments and aromatic/aliphatic diisocyanates along with chain extenders as hard segments [1]. In general, TPUs are elastomers with a linear molecular structure and glass transition temperatures below 0 °C that exhibit excellent mechanical properties, high abrasion resistance, low-temperature flexibility, biocompatibility and a large processing window, for which thermoplastic polyurethanes are widely used in various applications that include insulating materials, biomedical devices, degradable implants, electronic appliances and sports equipment [2,3,4]. By simply adjusting the nature or the volume fraction of the components that constitute the soft and hard segments or by modifying the polymerization pathway, thermoplastic polyurethanes with a unique and wide portfolio of mechanical properties, flexibility and/or biocompatibility can be obtained, leading to a variety of polymers with versatile structures [5,6,7,8]. The flexibility of the physical properties of polyurethanes is usually assigned to their complex morphology with alternating hard and soft segments as elements of the polymeric backbone, which leads to a microphase-separated specific organization triggered by the thermodynamic incompatibility between the constituent soft and hard domains [9,10,11]. An innovative approach in the polyurethane field was the insertion of ionic sequences into these polymers, which led to the obtaining of polyurethane ionomers [12,13,14]. Although the amount of ionic groups in polyurethane backbone is low (less than 15 mol%) [15,16], their presence in the polymeric material determines the formation of additional ionic networks through strong interaction via Coulombic forces or hydrogen bonds, with an effect on the physical and rheological behaviour of the resulting ionomers [16,17]. Regardless on the nature of the charge (positive, negative or both), all types of polyurethane ionomers, namely cationomers (bearing quaternary ammonium groups), anionomers (with carboxylate, sulphonate or phosphate units) and zwitterionomers (sulphobetaine) have been reported in the literature [18,19,20,21]. The increased interest shown in these polymers is generated by the fact that besides the standard properties of polyurethanes, the presence of ionic sequences determines modifications in the phase structure of polyurethanes, with an impact on their physical and rheological characteristics and implicitly on their application potential [18,22].

The recent development of hybrid materials, composed of both inorganic and organic elements, was triggered by the finding that compared to the raw materials, the resulting hybrid composites proved to display an improvement of the mechanical, thermal, optical or electrical properties. Up to now, various inorganic nanoparticles (metals, metal oxides, clays, carbon nanotubes) have been successfully embedded in polyurethane matrices [19,23,24,25,26]. The enhancement of composite properties is conditioned by the homogeneous and complete distribution of the inorganic phase in the organic matrix, which favours the increase in the interfacial surface interactions in tandem with the optimization of the organic–inorganic interactions. However, this goal is more difficult to accomplish, given the inherent immiscibility between the inorganic and organic phases, so extensive research has been devoted to the chemical modification of inorganic nanoparticles to improve the compatibility and implicitly the performance and applications of polyurethane composites [27,28,29]. Titanium dioxide (TiO_2_) nanoparticles have been frequently incorporated into polymer matrices used for applications in coatings, optical devices, photocatalysis, wastewater treatment or UV shielding due to their excellent chemical stability, optical properties, UV absorption, low cost and environmentally friendly nature [29,30,31]. It is well documented that TiO_2_ nanoparticles (NPs) in the anatase phase have a high refractive index and band gap energy of 3.2 eV with a low absorption coefficient in the visible range [32,33] and, depending on the envisaged application, can function as UV absorbers or photocatalysts due to their versatile behaviour in the presence of light tuned by the composition of the surrounding matrix [34]. Numerous studies have reported the preparation of transparent nanocomposites assembled of polymer films and inorganic nanoparticles (amount of 1–10 wt.%) displaying UV-shielding properties attributed to UV light absorption or UV light scattering phenomena [35,36], observing that a key element in the obtaining of the desired properties is represented by the degree of dispersion of the inorganic nanoparticles inside the polymer matrix.

In view of all these facts, this study reports the synthesis of new polyurethane elastomers with a variable soft-segment composition, along with the preparation of TiO_2_/polyurethane nanocomposites, by the quaternization of tertiary amine groups from polyurethane with silane-modified TiO_2_ nanoparticles. Thus, by the covalent immobilization of the functionalized nanoparticles in the polymer matrix, a controlled dispersion of TiO_2_ NPs could be achieved, while the presence of ionic sequences could enhance the interfacial interactions inside the composites, with an effect on the physicochemical and mechanical properties of the resulting coatings. In addition, different quaternization degrees were considered in order to investigate the influence of TiO_2_ loadings on the properties of the formed polymer nanocomposites.

## 2. Materials and Methods

### 2.1. Materials

Polycaprolactonediol (PCL) (average M_n_~1250 g/mol), poly(ethylene glycol) (PEG) (average M_n_~1000 g/mol), isophorone diisocyanate (IPDI, 98%), *N*-methyldiethanolamine (NMDA, 99%), 1,4-butanediol (1,4-BD, 99%), dibutyltin dilaurate (95%), titanium (IV) oxide, anatase (TiO_2_, 99.8%), (3-iodopropyl) trimethoxysilane (IPTMS, ≥95.0%), tetrahydrofuran (THF) and toluene (≥99.9%) were purchased from Sigma-Aldrich Chemical Co. (Taufkirchen, Germany) and used without further purification.

### 2.2. Synthesis of Elastomeric Polyurethanes

For the synthesis of PU-1 and PU-2 polyurethanes, the same general technique was employed, for the synthetic details given below refer to the preparation of the PU-1 polyurethane. First, 10 g (8 mmol) of PCL was degassed under vacuum for 2 h at 90 °C into a reaction kettle equipped with a mechanical stirrer, reflux condenser, dropping funnel and N_2_ inlet. Then, 5.2 mL (24 mmol) of IPDI and a few drops of catalyst dibutyltin dilaurate were added and the mixture was stirred at 60 °C for 3 h. Finally, 0.95 g (8 mmol) of NMDA and 0.72 g (8 mmol) of 1,4-BD dissolved in 20 mL anhydrous toluene were added and the reaction was continued for 10 h. The total disappearance of the isocyanate stretching band at 2260 cm^−1^ from the Fourier transform infrared (FTIR) spectrum confirmed the end of the reaction. For the preparation of the PU-2 polyurethane, a mixture of 8 g (6.4 mmol) of PCL and 1.6 g (1.6 mmol) of PEG was dehydrated under vacuum, the other steps being kept unchanged. The resulting polyurethanes (PU-1 and PU-2) were precipitated in methanol and dried under vacuum for 48 h.

**PU-1**: ^1^H NMR [CDCl_3_, δ ppm]: 4.21 (–O–CH_2_–C**H**_2_–O–CO– and NH–COO–C**H**_2_–CH_2_–N); 4.04 (–CO–O–C**H**_2_–CH_2_– from PCL and NH–COO–C**H**_2_–CH_2_–CH_2_–); 3.68 (–O–C**H**_2_–CH_2_– and >C**H**–NH–CO); 2.89 (–O–CO–NH–C**H**_2_–); 2.29 (–O–CO–C**H**_2_–CH_2_– from PCL and >N–C**H**_3_); 1.63 (–CO–O–CH_2_–C**H**_2_– and –O–CO–CH_2_–C**H**_2_– from PCL), 1.37, 1.04, 0.92, 0.87 (aliphatic protons from IPDI, PCL and BD). FTIR (KBr, cm^−1^): 3365 (NH); 2866–2956 (CH_2_); 1699–1738 (C=O); 1532 (amide II); 1237 and 1164 (C–O). M_n_ (g/mol) = 42,600; PDI: 2.57; yield: 91.5%.

**PU-2:**^1^HNMR [CDCl_3_, δ ppm]: 4.22 (–O–CH_2_–C**H**_2_–O–CO– and NH–COO–C**H**_2_–CH_2_–N); 4.05 (–CO–O–C**H**_2_–CH_2_– from PCL and NH–COO–C**H**_2_–CH_2_–CH_2_–); 3.68 (–O–C**H**_2_–CH_2_– and >C**H**–NH–CO); 3.63 (–O–C**H**_2_–C**H**_2_–O– from PEO); 2.89 (–O–CO–NH–C**H**_2_–); 2.29 (–O–CO–C**H**_2_–CH_2_– from PCL and >N–C**H**_3_); 1.63 (–CO–O–CH_2_–C**H**_2_– and –O–CO–CH_2_–C**H**_2_– from PCL), 1.37, 1.05, 0.92, 0.87 (aliphatic protons from IPDI, PCL and BD). FTIR (KBr, cm^−1^): 3343 (NH); 2868–2949 (CH_2_); 1730 (C=O); 1533 (amide II); 1238 and 1161 (C–O). M_n_ (g/mol) = 50,100; PDI: 2.21; yield: 93.1%.

### 2.3. TiO_2_ Nanoparticle Functionalization

The functionalization of TiO_2_ nanoparticles took place in two steps, according to a previously reported procedure [37]. Initially, 1.00 g of anhydrous TiO_2_ (12.5 mmol) and 0.1 mL of triethanolamine were mixed in ethanol (50 mL) to produce a homogeneous suspension. After heating to 60 °C, ammonium hydroxide (60 mL) and 1.5 mL of deionized water were injected into the flask, maintaining vigorous magnetic stirring. Further, the functionalization agent (3-iodopropyl) trimethoxysilane (IPTMS) (0.78 mL, 4.0 mmol) was added dropwise. The reaction mixture was maintained at 50 °C for 12 h and then was cooled to room temperature. The final nanoparticles were isolated by centrifugation after three cycles of purification (repeating washing/centrifugation procedures) to remove the excess of IPTMS. White-colour, functionalized TiO_2_ nanoparticles were obtained.

### 2.4. Quaternization of PU-1/PU-2 with Functionalized TiO_2_ Nanoparticles in THF

Modified polyurethane compounds containing quaternized ammonium groups were prepared by the use of two different molar ratios (1/1 and 1/0.5 molar ratios) of polyurethanes/functionalized TiO_2_. For example, 1 g of the PU-1 polyurethane (0.45 mmol) was stirred in 30 mL of THF until solubilization. Modified TiO_2_ nanoparticles (0.17 g, 0.45 mmol) were further added to the flask, and the reaction mixture was maintained at 40 °C for 72 h. Drop-casting flexible and homogeneous films were obtained after complete solvent evaporation. The same experimental procedure was maintained for all polyurethanes and compositions. For a better view, the molar ratios of the reagents introduced in the synthesis of polyurethanes and composites are given Table 1.

### 2.5. Measurements

Fourier transform infrared (FTIR) spectra were registered on a Bruker Vertex 70 FTIR instrument. Analyses were performed using KBr pellets in transmission mode in the range of 400–4000 cm^−1^ at room temperature with a resolution of 2 cm^−1^. The ^1^H NMR spectra were recorded on a BRUKER Avance DRX 400 spectrometer using CDCl_3_ as a solvent. The molecular weight and polydispersity index (PDI) of PU-1 and PU-2 polyurethanes was determined by gel permeation chromatography (GPC) in chloroform solution at 25 °C and at a flow rate of 1 mL min^−1^ using a WGE SEC/GPC multidetection chromatograph equipped with two Agilent PL gel 5 μm columns, MIXED D and MIXED C, capable of separating molecular weights within 200 to 2,000,000 g mol^−1^. Wide-angle X-ray diffraction (XRD) patterns for unmodified TiO_2_ nanoparticles, organically modified TiO_2_ and polyurethane/TiO_2_ hybrid composites were recorded via X-ray diffraction (Rigaku Miniflex 600 diffractometer) using CuKα radiation (λ = 0.154 nm) at ambient temperature in the angular range of 3°–90° (2θ) with a scanning step of 0.01° and a recording rate of 2°/min. The diffraction peaks were identified using the Crystallography Open Database (COD) data and the SmartLab II v. 4 software package for powder X-ray diffraction analysis. Scanning electron microscopy (SEM) and energy-dispersive X-ray spectroscopy (EDX) analyses were performed on aQUANTA200 environmental scanning electron microscope coupled with an energy-dispersive X-ray spectroscope (ESEM/EDX), operating at 20 kV in low-vacuum mode and using a Low vacuum Secondary Electron (LFD) detector. The SEM and EDX investigations were realized in a cryogenically broken fracture surface and in different points of the sample to check their reproducibility.

The stability of the polyurethane films (PU-1, PU-2) and of their corresponding composites (PU-1-0.5, PU-1-1.0, PU-2-0.5, PU-2-1.0) was tested as follows: a piece of each sample (100 mg) was immersed in 10 mL solution with different pH values: pH = 4 (Merck KGaA buffer solution citric acid/sodium hydroxide/hydrogen chloride), pH = 5.6 (distilled water), pH = 7 (Merck KGaA buffer solution potassium dihydrogen phosphate/di-sodium hydrogen phosphate) and pH = 9 (Merck KGaA buffer solution boric acid/potassium chloride/sodium hydroxide). All solutions with the films inside were heated at 60 °C and kept at this temperature for 3 h. Further, the solutions were cooled on an ice bath, and the films were removed from the solutions, left to dry in air for 2 h and then dried in an oven at 60 °C for 2 h. Finally, the films were weighed, and the procedure was repeated 7 times.

Static water contact angle measurements were obtained by using the sessile drop technique with a contact angle instrument (KSV CAM 101 goniometer—KSV Instruments, Helsinki, Finland), equipped with a special optical system and a charge-coupled device (CCD) camera. A drop of liquid (~1 μL) was placed on the films’ surfaces with a Hamilton syringe, and three different regions were selected to obtain a statistical result. Double distilled water and ethylene glycol were used as measuring liquids. The analyses were performed at room temperature, and the maximum error in the contact angle measurement did not exceed 2%. The thermal behaviour of polymeric materials was evaluated by thermogravimetric analysis (TGA) on a STA 449 F1 Jupiter apparatus (Netzsch, Selb, Germany). The measurements were performed in the temperature range of 25–700 °C under a dry nitrogen atmosphere at a heating rate of 10 °C·min^−1^. Sample weights in the range of 7–10 mg and Al_2_O_3_ crucibles were used.

Mechanical parameters of the polyurethane samples were investigated on a Shimadzu AGS-J deformation apparatus at ambient temperature and at a rate of deformation of 20 mm/min with a load cell capable of measuring forces up to 1 kN and a sample film of 25 mm × 5 mm × 0.3 mm. For each data point, five samples were tested, and the average value was taken. The dynamic mechanical properties of the samples were measured using a PerkinElmer Diamond dynamic mechanical analyzer DMA in tension mode operated at a frequency of 1 Hz, a heating rate of 2 °C min^−1^ and a temperature range of −100 °C to 55 °C. The samples used in the study were rectangular with a length of 10.0 mm, width of 5.0 mm and thickness of 0.2 mm. The UV–VIS absorption and transmittance spectra of the polyurethane films were measured using a Perkin Elmer Lambda 2 UV–VIS spectrophotometer (Perkin Elmer Inc., Wellesley, MA, USA) in the wavelength region of 200–700 nm. The UV light stability of polyurethanes (PU-1, PU-2) and composites (PU-1-0.5, PU-1-1.0, PU-2-0.5, PU-2-1.0) was evaluated by the exposure of the films to UV radiation with a light intensity of 15 mW/cm^2^ (Hg–Xe lamp, λ = 365 nm, Hamamatsu Lightningcure Type LC8, Model L9588, Iwata City, Shizuoka Pref., Japan). The transmittance spectra of the coatings were measured before and after different UV exposure time intervals.

## 3. Results and Discussion

### 3.1. Functionalization and Characterization of TiO_2_ NPs

**Figure 1:**
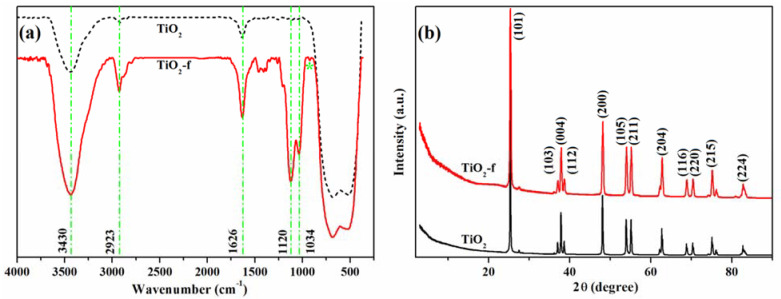
FTIR spectra (**a**) and XRD diffractograms (**b**) of the commercial TiO_2_ and functionalized TiO_2_ NPs.

Like other functionalization reactions, the aim of TiO_2_ surface modification was to improve the specific properties of inorganic nanoparticles regarding their hydrophilicity/hydrophobicity, dispersibility in different solvents, superior photocatalytic efficiency or, in our case, an enhanced dispersion and covalent attachment of TiO_2_ nanoparticles inside the polyurethane matrix. The effectiveness of titania functionalization was confirmed thorough FTIR spectroscopy by tracking the characteristic absorption bands of the new compound (Figure 1a).

Therefore, besides a broad Ti–O–Ti absorption band (400–700 cm^−1^), the OH groups’ absorption band (3430 cm^−1^) or molecular water (3430 and 1626 cm^−1^), the new TiO_2_ functionalized nanoparticles presented the Si–O–Si asymmetric stretching vibration at 1120 and 1034 cm^−1^ along with the intensification of the methylene groups’ characteristic band (2923 cm^−1^). The absorption band due to the vibration of Ti–O–Si new bonds was identified as a small shoulder at 922 cm^−1^ [38].

The X–ray diffraction patterns of the commercial and functionalized TiO_2_ NPs are illustrated in Figure 1b. The analysis of the spectra indicated that the pristine TiO_2_ nanoparticles are in the anatase structure (peaks identified according to the TiO_2_ Anatase COD card number 9015929), information confirmed by the presence of strong diffraction peaks at 25.37° and 48.1° specific to the anatase phase [39,40]. The crystallinity of the sample is reflected by the intense XRD peaks, and the crystallite size calculated from the diffraction peaks using the Debye–Scherer formula is about 45 nm. As expected, after modification with IPTMS, TiO_2_-f nanoparticles showed a similar pattern as the starting TiO_2_, since no phase transformation or changes in the crystalline structure took place during the functionalization.

### 3.2. Synthesis and Characterization of Polyurethane/TiO_2_ Composites

**Figure 2:**
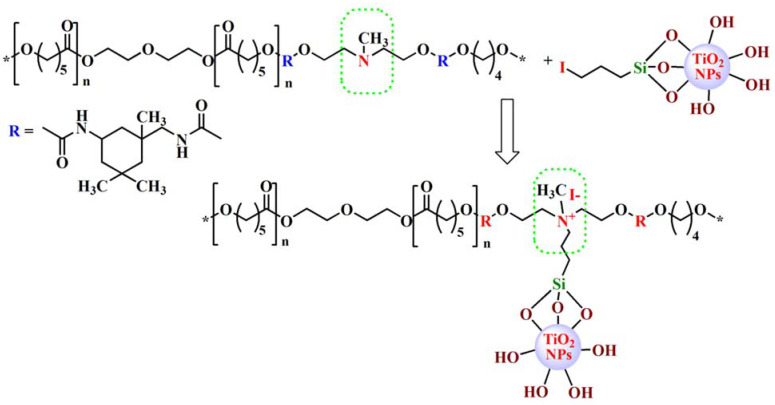
Representation of quaternization reaction between PU-1 and TiO_2_-f nanoparticles.

To obtain new hybrid materials based on polyurethane matrices and functionalized TiO_2_ nanoparticles, the quaternization of tertiary amino units from polyurethanes with iodide-modified TiO_2_ NPs was performed (Figure 2), aiming at achieving a covalent bonding between the organic and inorganic components, which subsequently could lead to polymer composites with improved properties. In the preparation of the materials, the functionalized nanoparticles were mixed in 1/1 and 1/0.5 molar ratios with polyurethane solutions in THF, which allowed the obtaining of four composites, namely PU-1-0.5, PU-1-1.0, PU-2-0.5 and PU-2-1.0 (initial molar compositions are given in Table 1).

**Figure 3:**
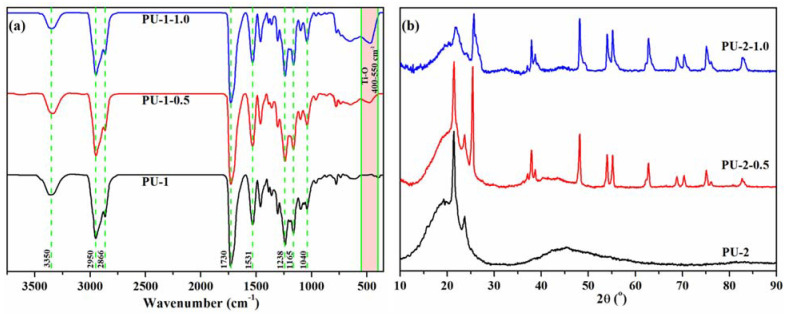
FTIR spectra of PU-1, PU-1-0.5 and PU-1-1.0 sample (**a**) and XRD patterns (**b**) for PU-2, PU-2-0.5 and PU-2-1.0 films.

The structural, morphological, thermal and mechanical properties of the resulting composites were investigated comparatively with the pristine polyurethanes. In the FTIR spectra of PU-1, PU-1-0.5 and PU-1-1.0 samples, illustrated in Figure 3a, a board absorption peak in the region of 3200–3500 cm^−1^ was attributed to the stretching vibration of N–H units from the urethane groups, while the absorption band of C=O units is evident at 1730 cm^−1^. The absorption bands centred at 2950 cm^−1^ and 2866 cm^−1^ are associated with the C–H symmetric and asymmetric stretching vibrations of the aliphatic –CH_2_– groups, the peak at 1531 cm^−1^ is attributed to the amide II vibration, while the peaks at 1238 cm^−1^ and 1165 cm^−1^ are given by the stretching vibration of the C–O–C linkages. The contribution of inorganic sequences is slightly visible on the peak at 1040 cm^−1^, where the Si–O–Si stretching vibration is noticeable, although the initial PU–1 polyurethane has a peak in the same region [41], attributed also to the stretching of C–O–C bonds in polyurethanes. Significant changes in the investigated FTIR spectra are evident in 755–400 cm^−1^, where the contribution of TiO_2_ NPs is exhibited by the appearance of a broad absorption band attributed to Ti–O–Ti vibrations.

The XRD profiles of the PU-2 polyurethane and PU-2-0.5/PU-2-1.0 nanocomposites are shown in Figure 3b. The XRD pattern of the PU-2 sample showed a broad peak in the range of 15°–25°, confirming the amorphous nature of the polyurethane, although the sharp peaks at 21.4° and 23.7° are given by the orthorhombic crystalline domains of PCL and can be assigned to the (110) and (200) lattice planes [42]. For the PU-2-0.5 composite, the XRD diffraction pattern has a similar profile as the pristine polyurethane in the 10°–24° domain, while above these values, the diffraction peaks characteristic to TiO_2_ nanoparticles are visible. However, in the case of the PU-2-1.0 sample, the peaks corresponding to crystalline PCL sequences are considerably diminished, the broad diffraction peak suggesting the presence of a semi-crystalline or mostly amorphous polymer. It can be assumed that the high amount of TiO_2_ NPs included in the PU-2-1.0 composite (about 17 wt.%) could hinder the crystallization pattern of PCL chains, leading to a lower crystallinity of the polymer. In addition, the peak at 25.38° specific to the anatase TiO_2_ (110) plane, is slightly shifted to 25.68° in the PU-2-1.0 sample, indicating an enhanced interaction with the polymer chains [43].

**Figure 4:**
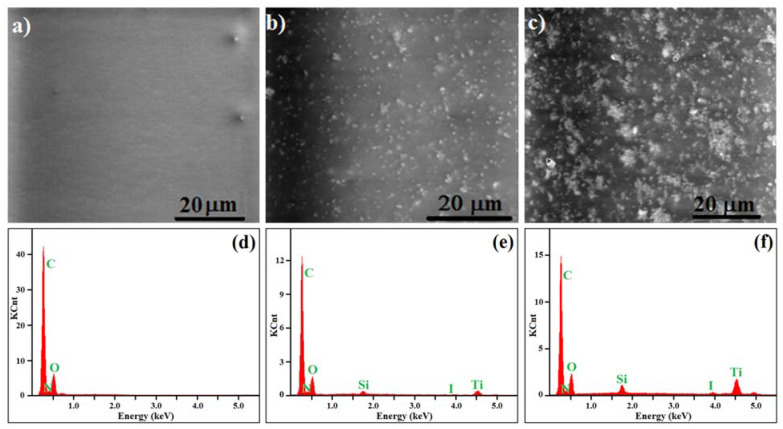
SEM microphotographs and the corresponding EDX measurements of the PU-2 polyurethane (**a**,**d**), PU-2-0.5 (**b**,**e**) and PU-2-1.0(**c**,**f**) composites films.

Scanning electron microscopy (SEM) images were taken to investigate the micro-morphology of the prepared polyurethane-based films with a different molar ratio of TiO_2_ nanoparticles in the blends (Figure 4). All photographs were taken on the fractured films’ surfaces at room temperature. As expected, the neat PU-2 film (Figure 4a) was characterized by a smooth, flat and compact network structure, without irregular formations due to the incompatibility of the polyurethane components. Instead, the presence of TiO_2_ particles was clearly observed in the SEM images for the PU-2-0.5 composite (Figure 4b). Moreover, the particles seemed to be uniformly dispersed inside the polyurethane matrix, which provides direct evidence regarding the micro-structure and the formation of PU-2-0.5 nanocomposites. Moreover, this homogeneity in the dispersion of the TiO_2_ nanoparticles in the polyurethane matrix will certainly help to improve the mechanical properties of the prepared blends.

However, for PU-2 nanocomposites with a higher TiO_2_ content (1:1 molar ratio, see PU-2-1.0), some small particle clusters appeared (Figure 4c), in addition to the homogeneously distributed primary nanoparticles. It seems like a high percentage of the inorganic component does not particularly help to form a film with a uniform distribution of particles, suggesting, moreover, that the interfacial interaction between the constituents is not so strong in order to suppress the formation of aggregates. In addition, the presence of TiO_2_ nanoparticles in the micro- and nanostructures of PU-2-0.5 and PU-2-1.0 composite films was confirmed by EDX measurements performed on randomly selected areas of the cross sections of the films at the same time as SEM imaging (see Figure 4). As expected, the results indicated that the elements of C, O, Si, I and Ti were present on the surface of composite films (Figure 4e,f) but only C, N and O were found on the surface of pure PU-2 replicas (Figure 4d). Moreover, the atomic content of Si and Ti elements registered for PU-2-1.0 is higher with respect to the PU-2-0.5 formulation, in accordance with the content of TiO_2_ nanoparticles embedded in each composite.

The thermal/hydrolytic stability of polymers represents an important parameter, especially for systems intended to be applied in medical, biomedical or food domains, and numerous protocols of testing were proposed in the literature [44]. In our study, to assess the films’ stability at different pH values and under repetitive heating–cooling cycles, only some preliminary tests were performed by measuring the samples’ weights before and after each heating–cooling cycle of the films immersed in solution at different pH values (4, 5.6, 7 and 9). The polyurethane films (PU-1, PU-2) and polyurethane/TiO_2_ composites (PU-1-0.5, PU-1-1.0, PU-2-0.5, PU-2-1.0) displayed good hydrolytic stability, and the investigated samples did not show weight loss after seven heating–cooling cycles in solutions with different pH values.

**Figure 5:**
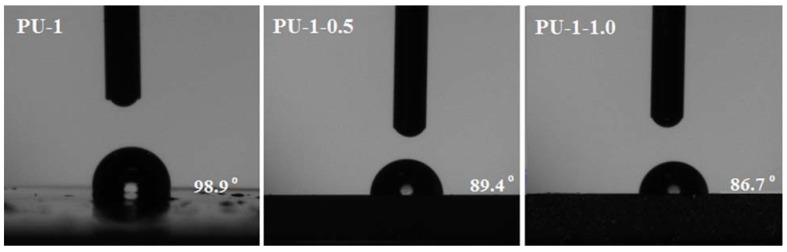
Static contact angle measurements for the neat PU-1 film surface and the corresponding composites (PU-1-0.5 and PU-1-1.0) at room temperature.

The contact angle measurements have commonly been employed as a simple method to evaluate the surface properties of polyurethane films, especially wettability/hydrophobicity properties. Generally, the contact angle (θ) is recognized as the angle formed between the baseline and the tangent to the liquid drop at the three-phase point [45]. By convention, all surfaces having a contact angle greater than 90° are defined as being hydrophobic, while surfaces with values smaller than 90° are considered hydrophilic ones. According to the above information, the contact angle data shown in Figure 5 indicate the tendency of the surfaces to change their nature from a predominantly hydrophobic (neat PU-1 film, contact angle 98.8°) to a slightly hydrophilic one (89.4° for PU-1-0.5 and 86.7° for PU-1-1.0).

This behaviour is the result of changes in the composition of the polyurethane composites, more precisely the introduction of the inorganic filler (TiO_2_ nanoparticles) to the polyurethane matrix. The neat PU-1 film exhibited an average contact angle of 98.9° (suggesting a hydrophobic surface), and the value decreased to 89.4° (PU-1-0.5) and 86.7° (PU-1-1.0). As mentioned, this trend indicated an increase in hydrophilicity (better wettability) of the film surface for the nanocomposites compared to the neat polyurethanes, more probably due to arising of the polar sequences (hydroxyl from the TiO_2_ surface or ionic units), which determines an increase in the surfaces’ hydrophilicity [46,47]. Moreover, these results are correlated with the content of the filler: the higher the amount of inorganic material added to the composite, the lower the value of the contact angle. Similar results were observed when ethylene glycol was used to determine the contact angle, too.

**Figure 6:**
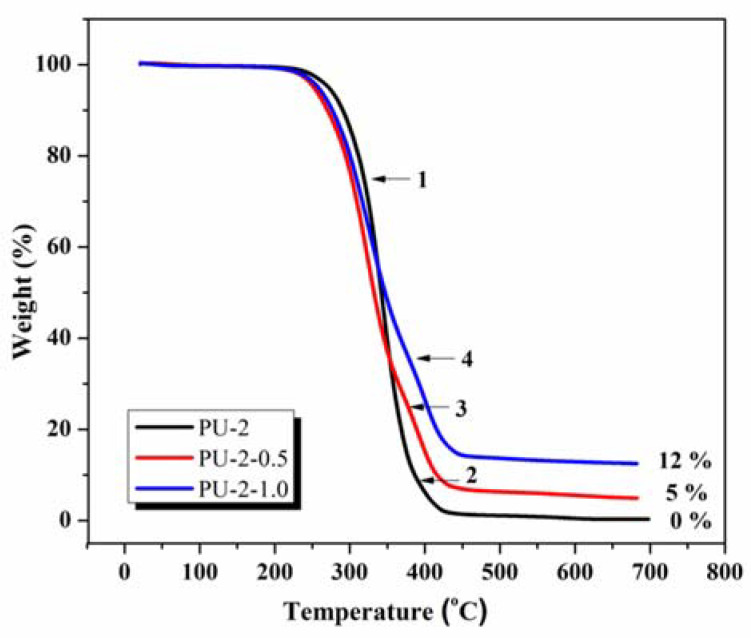
TGA thermograms for the neat PU-2 polyurethane and the corresponding composites PU-2-0.5 and PU-2-1.0.

TGA experiments were also performed in this study in order to determine the thermal stability of the neat PU-2 material and PU-2/TiO_2_nanocomposites (with different molar ratios of TiO_2_). Generally, the polyurethanes’ thermograms refer to a complex decomposition, usually in two degradation stages: the process starts with the scission of the hard segment and continues with the degradation of the soft segment in a second threshold. According to the literature data [48,49], the first TGA peak is attributed to the overlapping of urethane bond degradation and char-forming secondary reactions (e.g., dimerization, crosslinking, etc.). At this level, the breaking of low-energy urethane bonding with the release of CO, CO_2_, amines and aldehydes takes place. At the same time, the second peak is associated with the decomposition of the stabilized urea/isocyanurate structures and is related to the breaking of high-energy bonds, such as C–C, C–O, C–H, C=C and C=O. Given all this information, Figure 6 presents the thermogravimetric curves of the studied PU-2 and PU-2/TiO_2_nanocomposite materials. As observed, two main degradation/decomposition stages were evidenced: a first one between 220 and 350 °C (point **1**) and a second one around 350–420 °C (points **2**, **3** and **4**). The TGA curve for the neat PU-2 presented a clear stage for the degradation of the urethane linkage, with a T_max_ = 332 °C, while the step corresponding to the degradation of the soft segment was not so prominent and could be spotted around 392 °C.

The equivalent nanocomposite materials (PU-2-0.5 and PU-2-1.0) showed essentially similar TGA profiles, but the degradation stages were more distinct compared to the neat PU-2 case. The process began with the scission of the hard segment (at an inferior temperature than the neat polyurethane) and continued with the degradation of the softsegment in a second distinct threshold. As observed, the first stage of decomposition began at a lower temperature, but this is not necessarily synonymous with a low thermal stability of the systems. Most probably, this is due to the removal of volatile components or other low-molecular-weight materials that arise after the corresponding functionalization process of the TiO_2_ nanoparticles. Moreover, PU-2-0.5 and PU-2-1.0 composites showed a second decomposition peak shifted to a higher temperature range with 10–20 °C than in the case of the starting PU, probably associated with a strong interfacial interaction between the polyurethane matrix and TiO_2_ nanoparticles. Consequently, this feature indicates improved thermal stability of the systems due to the presence of the inorganic component. After 500 °C, no mass loss was observed, meaning that the mass of the materials remained constant, and probably only changes in the crystal structure of the inorganic component were expected. Furthermore, at 700 °C, the starting PU-2 presented no mass residue, while for nanocomposites, the percentage of residues increased from 5% to 12% with the augmentation of the molar ratio between the components for PU-2-0.5 and PU-2-1.0, respectively.

**Figure 7:**
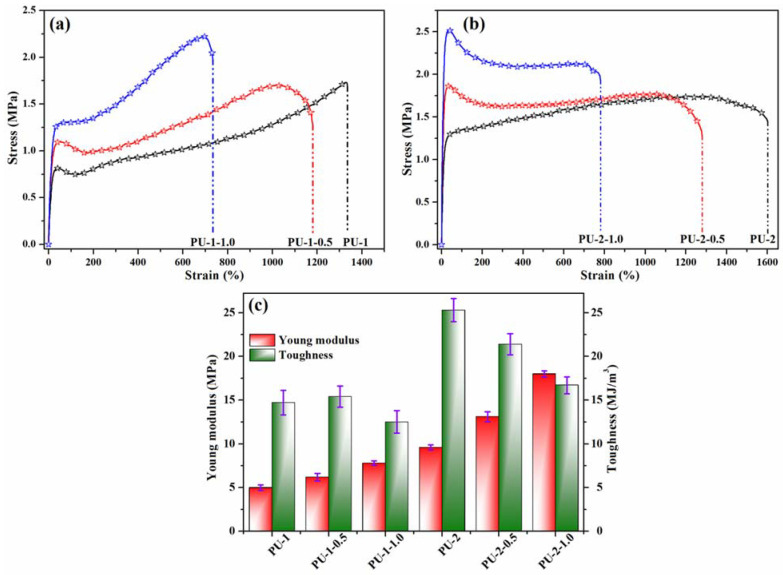
Representative stress–strain curves for polyurethanes and composite specimens (**a**,**b**) and the statistical results of the Young modulus and toughness measurements (**c**).

The mechanical properties of polyurethanes and composite samples were measured by tensile testing, and the achieved results are illustrated in Figure 7. The stress–strain curves of PU-1 and PU-2 polyurethanes suggest an elastomeric behaviour since the maximum tensile strength is 1.73 MPa for PU-1 and 1.74 MPa for PU-2, while the elongation at break is 1335% for PU-1 and 1600% for PU-2, indicating the obtaining of highly flexible materials (Figure 7a,b). However, the shape of the curves is slightly different, as the tensile curve of the PU-1 polyurethane containing only PCL polyol displays a yield point, followed by an ascending shape of the graph, while PU-2 does not exhibit a yield point, which demonstrates better elastomeric properties that can be attributed to the PCL/PEG polyol mixture. The inclusion of functionalized TiO_2_ NPs through quaternization on polyurethane chains determines the formation of yield points in all composites, accompanied by a decrease in elongation at break, suggesting the stiffening of polymer chains.

However, despite the large amount of NPs introduced, polyurethane composites with strains at a break above 780% were achieved. The Young modulus evaluated from the low-strain region of the stress–strain curves varied between 5.0 MPa for PU-1 and 18.1 MPa for PU-2-1.0 (Figure 7c). In both polymer series, an increase of Young modulus values with increasing TiO_2_ nanoparticle content can be noticed, the filler particles behaving as reinforcing points in the polyurethane structure.

Furthermore, the toughness, another important mechanical property that characterize the ability of a material to absorb energy and plastically deform without fracturing and defined as the area under the stress–strain curve before rupture, was evaluated, and the results are graphically illustrated in Figure 7c. Toughness variation in the case of the PU-1 series is low (±10%), suggesting good dispersion and enhanced interconnectivity between the polyurethane and TiO_2_ nanoparticles [50], preserving thus the ductility of the resulting composites. However, in the PU-2 series, the composite toughness decreases by about 15% for PU-2-0.5, while for the PU-2-1.0 sample, the toughness decreased to a greater extent (about 35%), which may suggest the agglomeration of some TiO_2_ NPs that may act as defects and reduce the ductility of the composite.

**Figure 8:**
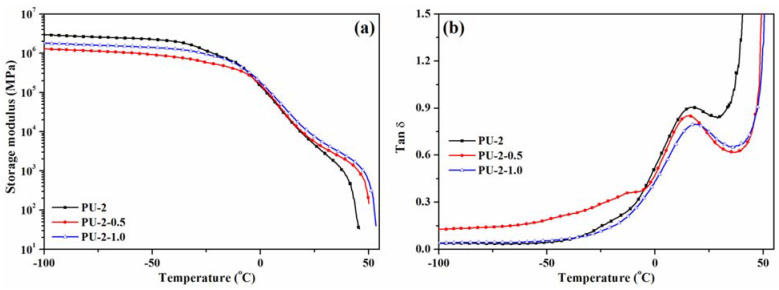
Storage modulus (**a**) and damping ratio (**b**) of PU-2, PU-2-0.5 and PU-2-1.0 samples as a function of temperature under a DMA loading frequency of 1 Hz.

A preliminary study concerning the dynamic mechanical properties of the PU-2 polyurethane and PU-2-0.5/PU-2-1.0 composites was performed in order to establish the influence of TiO_2_ nanoparticles on the storage modulus (*E*′) and tan delta (*T_g_)* parameters. The results of DMA tests, performed in the temperature range of −100÷55 °C, are graphically illustrated in Figure 8. The storage modulus (*E*′) represents the contribution of the elastic component of the films, and as can be observed from Figure 8a, in the glassy region (−100÷−30 °C), the storage modulus values for the PU-2 sample are higher than for the corresponding composites, a behaviour that may be assigned to the agglomeration of some inorganic nanoparticles [51]. However, in the rubbery region (25÷50 °C), the storage modulus increased for the filled coatings (PU-2-0.5 and PU-2-1.0) as compared to the neat polyurethane, implying that the presence of TiO_2_ nanoparticles caused an improvement of the modulus for the nanocomposite coatings. It should be noticed that given the high amount of TiO_2_ nanoparticles included in the polyurethane matrix, the differences in storage modulus are not significant over the entire temperature range. At temperatures above 50 °C, the storage modulus decreases to low values due to the irreversible polymeric chain flow triggered by the elastomeric character of polyurethanes [52], suggesting that the proposed nanocomposites may be successfully used at moderate temperatures.

Tan δ (loss factor/damping ratio), defined as the ratio of the loss modulus to the storage modulus, was also evaluated for the PU-2 series coatings (Figure 8b). It was observed that the pure PU-2 film showed a damping peak at 13.8 °C assigned to the T_g_ of the hard segment, while the peak position of tan δ shifted to a lower position for the PU-2-0.5 sample (12.3 °C) and to a higher value (16 °C) for the PU-2-1.0 sample. In addition, a reduction in the peak height was noticed for the nanocomposites as compared to neat polyurethanes, suggesting efficient stress transferring and a stronger nanoparticle/polymer interaction [53].

### 3.3. Optical and UV-Shielding Properties of Polyurethane/TiO_2_ Composites

**Figure 9:**
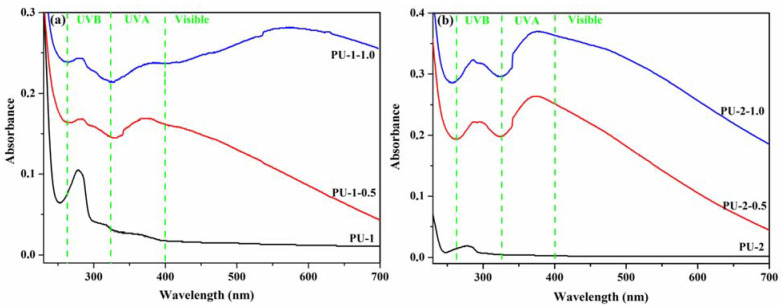
UV–VIS absorption spectra of the polyurethane nanocomposite films: PU-1, PU-1-0.5 and PU-1-1.0 (**a**) and PU-2, PU-2-0.5 and PU-2-1.0 (**b**).

The optical properties of pure PU-1, PU-2 films and TiO_2_/PU-1 andTiO_2_/PU-2 nanocomposite films were investigated through UV–VIS spectroscopy by measuring their UV–VIS absorption and transmittance spectra. The pure polyurethane films PU-1 and PU-2 show a weak absorption band at around 280 nm (Figure 9), in the UV-B radiation range from sunlight (280–320 nm). The incorporation of TiO_2_ nanoparticles in the polyurethane matrix led to the appearance of a new absorption band at 373 nm, corresponding to the UV-A radiation domain from sunlight (320–400 nm), indicating that polyurethane/TiO_2_ composite films have UV light absorption ability. In addition, it can be noticed that the composites with a higher TiO_2_ content (PU-1-1.0 and PU-2-1.0) have absorption bands with higher intensity; thus PU-1-1.0 and PU-2-1.0 are more efficient as UV absorbers.

**Figure 10:**
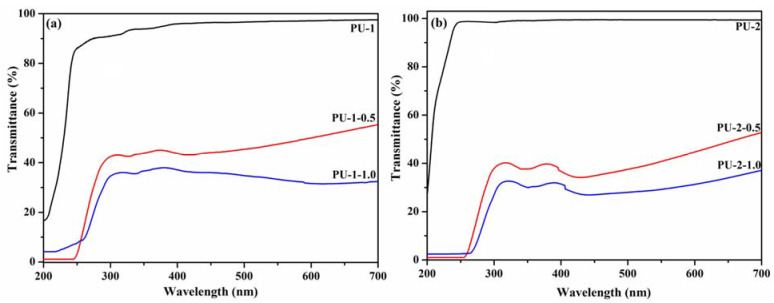
UV–VIS transmittance spectra of the polyurethane nanocomposite films: PU-1, PU-1-0.5 andPU-1-1.0 (**a**) and PU-2, PU-2-0.5 andPU-2-1.0 (**b**).

Moreover, analyzing the transmittance spectra (Figure 10), it was observed that the pure polyurethane films (50 μm thickness) display good transmittance in the range of 250–700 nm: 95% for PU-1 and 98% for PU-2. The incorporation of TiO_2_ nanoparticles in the PU-1 and PU-2 matrix confers UV-shielding properties to the materials, the composites PU-1-0.5, PU-1-1.0, PU-2-0.5 and PU-2-1.0 being able to shield all UV-B radiation (until 320 nm), as can be seen in Figure 10a,b. In addition, the composite films also shield part of the UV-A radiation, their transmittance measured at 350 nm being as follows: 39% for PU-1-0.5, 33% for PU-1-1.0, 43% for PU-2-0.5 and 37% for PU-2-1.0.

Even if the polyurethane/TiO_2_ composites lose part of their transparency to visible light (transmittance at 550 nm: 48% for PU-1-0.5, 35% for PU-1-1.0, 41% for PU-2-0.5, 29% for PU-2-1.0), they block between 57% and 67% of UV-A radiation and all UV-B radiation from sunlight; thus our materials are recommended as UV-protective coatings in various domains. Even if the particles are uniformly dispersed inside the polyurethane matrix, the transparency to visible light of the composite films is reduced due to the relatively large size of commercial TiO_2_ NPs. To preserve the transparency of the films in visible light, as already reported in the literature for various TiO_2_ coatings [54,55], it is recommended to use TiO_2_ nanoparticles with smaller dimensions, synthesized in the laboratory at the desired size, a topic that will be developed in a subsequent study. In this report, we investigated whether the presence of TiO_2_ nanoparticles affects polyurethane properties (thermal, mechanical) and whether they bring UV-shielding features to the final materials.

**Figure 11:**
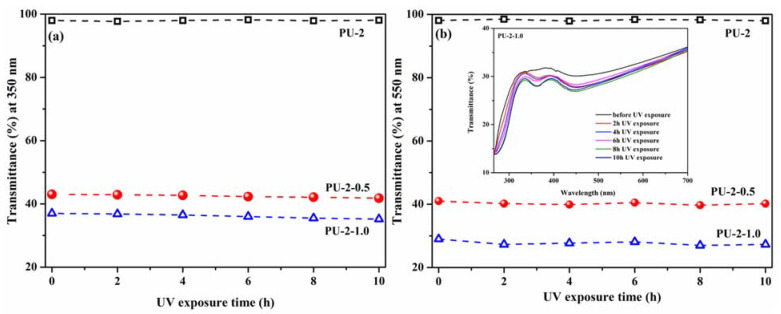
Transmittance values at 350 nm (**a**) and 550 nm (**b**) of PU-2, PU-2-0.5 and PU-2-1.0 films after different UV exposure time intervals. The inset is the transmittance spectra of PU-2-1.0 after different UV exposure time intervals (2, 4, 6, 8 and 10 h).

The literature data reported the preparation of numerous TiO_2_/polymer systems used in various chemical engineering processes [33], where chemical, thermal or mechanical stability is required. However, to verify whether the proposed films can be used as UV-protective coatings for a long period of time, some preliminary tests concerning the stability of the samples under UV radiation were performed. Each prepared film was exposed to UV light with the intensity of 15 mW/cm^2^ (which is the intensity of UV radiation from sunlight in the summer), and their transmittance spectra were registered after different UV exposure time intervals. After a total exposure time to UV radiation of 10 h, no significant changes were observed in the transmittance spectra, as presented in Figure 11 for the PU-2 film and composites.

The transmittance values of the films at 350 nm after 10 h of UV irradiation decreases with about 1.8%; meanwhile, the transmittance in the visible domain (at 550 nm) decreases with 1.5% after the first 2 h of irradiation, and then it is maintained at approximately the same value. Hence our films are stable under UV radiation, confirming once again that they can be used as UV-protective coatings for a long period of time.

## 4. Conclusions

Elastomeric polyurethane composites were prepared by the quaternization of tertiary amino units from polyurethanes with iodide-modified TiO_2_ NPs. The functionalization of the TiO_2_ surface with organic moieties ensures a homogeneous dispersion of TiO_2_ nanoparticles inside the polyurethane matrix, together with the covalent bonding between the organic and inorganic components. The inclusion of functionalized TiO_2_ NPs in the polyurethanes improved the thermal stability of the resulted systems. In terms of mechanical properties, the filler particles behave as reinforcing points in the polyurethane structure, and also, the composite materials preserve the high flexibility and ductility of the pure polyurethanes. TiO_2_/PU-1 andTiO_2_/PU-2 nanocomposite films are efficient UV absorbers, shielding between 57–67% of UV-A radiation and all UV-B radiation from sunlight. The prepared elastomeric materials are recommended as UV-protective coatings in various domains.

## Figures and Tables

**Table 1:** Initial feed ratio of materials in the preparation of polyurethanes and nanocomposites.

Sample	PCL (mol)	PEG (mol)	IPDI (mol)	NMDA (mol)	1,4-BD (mol)	TiO_2_-f	
						(mol)	(wt.%)
PU-1	1	0	3	1	1	0	0
PU-1-0.5	1	0	3	1	1	0.5	8.5
PU-1-1.0	1	0	3	1	1	1	17
PU-2	0.8	0.2	3	1	1	0	0
PU-2-0.5	0.8	0.2	3	1	1	0.5	8.5
PU-2-1.0	0.8	0.2	3	1	1	1	17

## Data Availability

Data are available on request.
